# Short-term Oral Antibiotics Treatment Promotes Inflammatory Activation of Colonic Invariant Natural Killer T and Conventional CD4^+^ T Cells

**DOI:** 10.3389/fmed.2018.00021

**Published:** 2018-02-07

**Authors:** Claudia Burrello, Federica Garavaglia, Fulvia Milena Cribiù, Giulia Ercoli, Silvano Bosari, Flavio Caprioli, Federica Facciotti

**Affiliations:** ^1^Department of Oncology and Hemato-Oncology, Università degli Studi di Milano, Milan, Italy; ^2^Department of Experimental Oncology, European Institute of Oncology, Milan, Italy; ^3^Pathology Unit, Fondazione IRCCS Cà Granda, Ospedale Policlinico di Milano, Milan, Italy; ^4^Gastroenterology and Endoscopy Unit, Fondazione IRCCS Cà Granda, Ospedale Maggiore Policlinico, Milan, Italy; ^5^Department of Pathophysiology and Transplantation, Università degli Studi di Milano, Milan, Italy

**Keywords:** iNKT cells, antibiotics, microbiota, T cells, intestinal inflammation

## Abstract

The gut mucosa is continuously exposed to a vast community of microorganisms, collectively defined as microbiota, establishing a mutualistic relationship with the host and contributing to shape the immune system. Gut microbiota is acquired at birth, and its composition is relatively stable during the entire adult life. Intestinal dysbiosis, defined as a microbial imbalance of gut bacterial communities, can be caused by several factors, including bacterial infections and antibiotic use, and has been associated with an increased risk to develop or exacerbate immune-mediated pathologies, such as allergic reactions, asthma, and inflammatory bowel diseases. Still, the mechanisms by which antibiotic-induced gut dysbiosis may lead to development of mucosal inflammation are still matter of debate. To this end, we aimed to evaluate the impact of antibiotic treatment on phenotype and functions of intestinal immune cell populations, including invariant natural killer T (iNKT) cells, a subset of lipid-specific T cells profoundly influenced by alterations on the commensal microbiota. To this aim, a cocktail of broad-spectrum antibiotics was administered for 2 weeks to otherwise healthy mice before re-colonization of the intestinal microbial community with oral gavage of eubiotic or dysbiotic mucosa-associated bacteria and luminal colonic content, followed or not by intestinal inflammation induction. Here. we showed that short-term antibiotic treatment alters frequency and functions of intestinal iNKT cells, even in the absence of intestinal inflammation. The presence of a dysbiotic microbiota after antibiotic treatment imprints colonic iNKT and CD4^+^ T cells toward a pro-inflammatory phenotype that collectively contributes to aggravate intestinal inflammation. Nonetheless, the inflammatory potential of the dysbiotic microbiota decreases over time opening the possibility to temporally intervene on the microbial composition to re-equilibrate dysbiosis, thus controlling concomitantly mucosal immune T cell activations.

## Introduction

The gut mucosa is a complex environment, constantly exposed to a vast community of microorganisms, collectively defined as microbiota, which include bacteria, fungi, protozoa, and viruses (1). Intestinal microbiota establishes a mutualistic relationship with the host, providing metabolic functions and contributing to shape the immune system (2). Indeed, germ-free mice (GF) manifest profound defects in the immune system development and function (3). Moreover, the presence of specific bacterial strains in the gut, such as segmented filamentous bacteria and *Clostridia* cluster IV and XIV, have been linked, respectively, to the differentiation and expansion of IL-17 producing CD4^+^Th17 cells (4) and of Foxp3^+^ regulatory T cells (5). To preserve intestinal immune homeostasis, active processes must thus operate, aimed at preserving the capacity of the gut-associated immune system to recognize invading pathogens and simultaneously avoiding immune responses against the commensal intestinal microbiota (1).

Intestinal dysbiosis, defined as a perturbation to the structure of intestinal commensal communities (6), can be triggered by several factors, including a persistent change in dietary habits, gastrointestinal infections, and alcohol misuse (7). Antibiotic administration can also lead to a profound perturbation of intestinal commensal communities, which persists after cessation of therapy, as a consequence of their broad-spectrum of action (8, 9). Clinical evidences support a correlation between antibiotic use and emergence or exacerbation of immune-mediated inflammation, as shown for atopic reactions (10), asthma (11), and inflammatory bowel diseases (IBD) (12). Importantly, several studies have consistently demonstrated that antibiotic use early in life predisposes to IBD emergence in Western populations (13).

Despite these evidences, the impact of antibiotic treatment and antibiotic-induced dysbiosis on distinct immune cells functions is still largely unexplored. In this context, few recent data indicate that neonatal mice treated with vancomycin or colistin manifest reduced intestinal lymphoid follicles, while broad-spectrum antibiotics administration diminishes antimicrobial peptides production (14). Vancomycin treatment additionally decreases Tregs colonic frequencies (15) while prolonged broad-spectrum antibiotics exposure induces intestinal and systemic alterations in the immune repertoire, including memory/effector T cells, Tregs, and dendritic cells (16). At present, however, it is unknown the effect of antibiotic treatment on frequency and functions of intestinal lipid-specific T cells such as iNKT cells.

Invariant natural killer T cells are a subset of CD1d-restricted αβ-T lymphocytes recognizing both self- and microbial-derived glycolipids (17–19) and showing both innate and adaptive immune characteristics (20). In line with data from conventional CD4^+^ T cells (4, 5, 15), increasing evidences support the existence of mutual mechanisms of regulation between the intestinal microbiota and iNKT cells (21). During early neonatal and postnatal stages of development, commensal bacteria negatively shape iNKT cell repertoire through a CXCL16-dependent gradient (22). Additionally, CD1d-dependent lipid antigens isolated from the commensal *B. fragilis* directly influence iNKT cell proliferation and activation status (23).

Here we evaluated the effect of antibiotics and antibiotic-induced microbiota alterations on colonic T cell immune responses, focusing specifically on iNKT cells. We tested the consequences of re-colonization of the gastrointestinal tract with normal or dysbiotic microbiota on iNKT cell phenotype and function and how it might translate into a specific outcome in the absence or presence of intestinal inflammation.

We provide evidences that antibiotic treatment in adult mice profoundly alters frequency and functions of intestinal iNKT cells even in the absence of intestinal inflammation, and that the presence of a dysbiotic microbiota after antibiotic treatment imprints colonic iNKT and conventional CD4^+^ T cells toward a pro-inflammatory phenotype that altogether contributes to aggravate intestinal inflammation. Nonetheless, the inflammatory potential of the dysbiotic microbiota decreases over time, opening the possibility to temporally intervene to re-equilibrate dysbiosis, thus controlling concomitantly mucosal immune cell activations.

## Materials and Methods

### Mice

C57BL/6 mice (Charles River, IT) and CXCR6-^EGFP/+^ mice (purchased as GFP/GFP from JAX, USA, and bred to heterozigosity with C57BL/6 mice) of 8–10 weeks of age were housed at the IEO animal facility in SPF conditions. Animal procedures were approved by the Italian Ministry of Health (Auth. 127/15, 27/13, 913/16).

### Experimental Colitis Models

For the induction of acute colitis, mice were given 2% (w/v) DSS (molecular weight 40 kD; TdB Consultancy) in their drinking water for 7 days followed by 2 days of recovery before sacrifice. The weight curve was determined by weighing mice daily. At sacrifice, colons were collected, their length was measured with a caliper, and then divided in portions to be fixed in 10% formalin for histological analyses, snap-frozen for RNA extraction, and for lamina propria mononuclear cells (LPMC) immunophenotyping.

### Antibiotic Treatment and Microbiome Reconstitution

To eliminate the gut microflora, mice were administered with a mix of neomycin (1 g/L), ampicillin (1 g/L), vancomycin (0.5 g/L), and metronidazole (1 g/L) in their drinking water. After 14 days of antibiotic treatment, mice received a transfer of mucus (first day) and feces (second and third days) from untreated (eubiotic) or DSS-treated (dysbiotic) donors by oral gavage. Mucus was scraped from donor colons, diluted in PBS, and administered to recipients at 1:1 ratio. Feces were collected from different donor mice, diluted in PBS (50 mg/mL), and administered to recipients by oral gavage. Mice were sacrificed at different time points according to the experimental settings. In some experiments after fecal microbiota transplantation (FMT), acute colitis was induced by administration of 2% DSS.

### Cell Isolation

For the isolation of LPMC from colons, Peyer’s patches were removed, lamina propria lymphocytes were isolated *via* incubation with 5 mM EDTA at 37°C for 30 min, followed by further digestion with collagenase IV and DNase at 37°C for 1 h. Cells were then separated with a Percoll density gradient (Sigma-Aldrich, St. Louis, MO, USA). Mesenteric LN and spleens were smashed into 70-µm nylon strainers (BD) and eritrocytes lysed with RBC Lysis buffer (BD). Livers were mechanically dissected and mononuclear cells separated with a Percoll density gradient (Sigma-Aldrich, St. Louis, MO, USA).

In some experiments, after isolation, cells were re-stimulated *in vitro* for 3 h with PMA/ionomycin in the presence of Brefeldin A to evaluate cytokine secretion.

### Flow Cytometry Analysis

Mouse iNKT cells were identified by CXCR6-^EGFP^ expression or by mCD1d:PBS57 Tetramer (NIH Tetramer core facility) staining. Murine cells were stained with combinations of directly conjugated antibodies: CD45.2 (104), CD3 (17A2) CD8α (53–6.7), CD4 (GK1.5), CD69 (H1.2F3), CD19 (1D3), CD11b (M1/70), F4/80 (BM8), Ly6g (1A8), Ly6c (AL-21), CD11c (HL3) all purchased from BD, eBioscience, or Biolegend. Gating strategy to identify T cells comprised the exclusion of CD11b^+^, CD19^+^, and CD11c^+^ cells (defined as “lineage”).

Intracellular staining of cytokines was performed according to standard methods. Cells were fixed and permeabilized with Cytofix/Cytoperm (BD) before addition of the following antibodies: anti-IFNg (XMG1.2), anti-IL17A (TC11-18H10.1), anti-IL10 (JES5-16E3), anti IL22 (Poly5164) (BD or eBioscience or Biolegend). Samples were analyzed by a FACSCanto flow cytometer (BD), gated to exclude nonviable cells. Data were analyzed using FlowJo software (Tristar).

### qPCR Protocol for Quantification of Tissue mRNA

Total RNA from mouse colonic tissues was isolated using TRIZOL and Quick-RNA MiniPrep (ZymoResearch) according to manufacturer’s instructions. cDNAs were generated from 1 µg of total RNA with reverse transcription kit (Promega). Gene expression levels were evaluated by qPCR and normalized to *Rpl32* gene expression. The primer sequences are collected in Table S1 in Supplementary Material.

### 16S qPCR Protocol for Quantification of Bacterial DNA

Total bacterial DNA was extracted from mouse feces with the G’NOME DNA extraction kit (MP Biomedicals) according to manufacturer’s specifications. Bacterial DNA was analyzed by qPCR using 16S rDNA primers (SIGMA) and collected in the Table S1 in Supplementary Material. The relative abundance of each bacterial group was normalized to Eubacteria using the 2^−ΔΔCt^ method.

### Histological Analysis

Tissue processing was performed with a LEICA PELORIS processor before paraffin embedding. Murine samples were included using an automated system (SAKURA Tissue-Tek). After Hematoxylin and Eosin staining, snapshots of histology were taken using an Aperio CS2 microscope with a scanning resolution of 50,000 pixels per inch (0.5 µm per pixel with 10× objective and 2.5 µm per pixel when scanning at 4×). Scoring of disease activity was performed according to the criteria described in Table S2 in Supplementary Material.

### Statistical Analysis

Statistical significance was calculated using Kruskal–Wallis nonparametric test for multiple comparisons or unpaired nonparametric Mann–Whitney *t*-test for comparisons between two groups. *P* < 0.05 (*), *P* < 0.01 (**) *P* < 0.001 (***) were regarded as statistically significant. Outliers detected with Grubb’s test.

## Results

### Antibiotic Treatment Influences Colonic iNKT Cell Frequency and Phenotype

**Figure 1:**
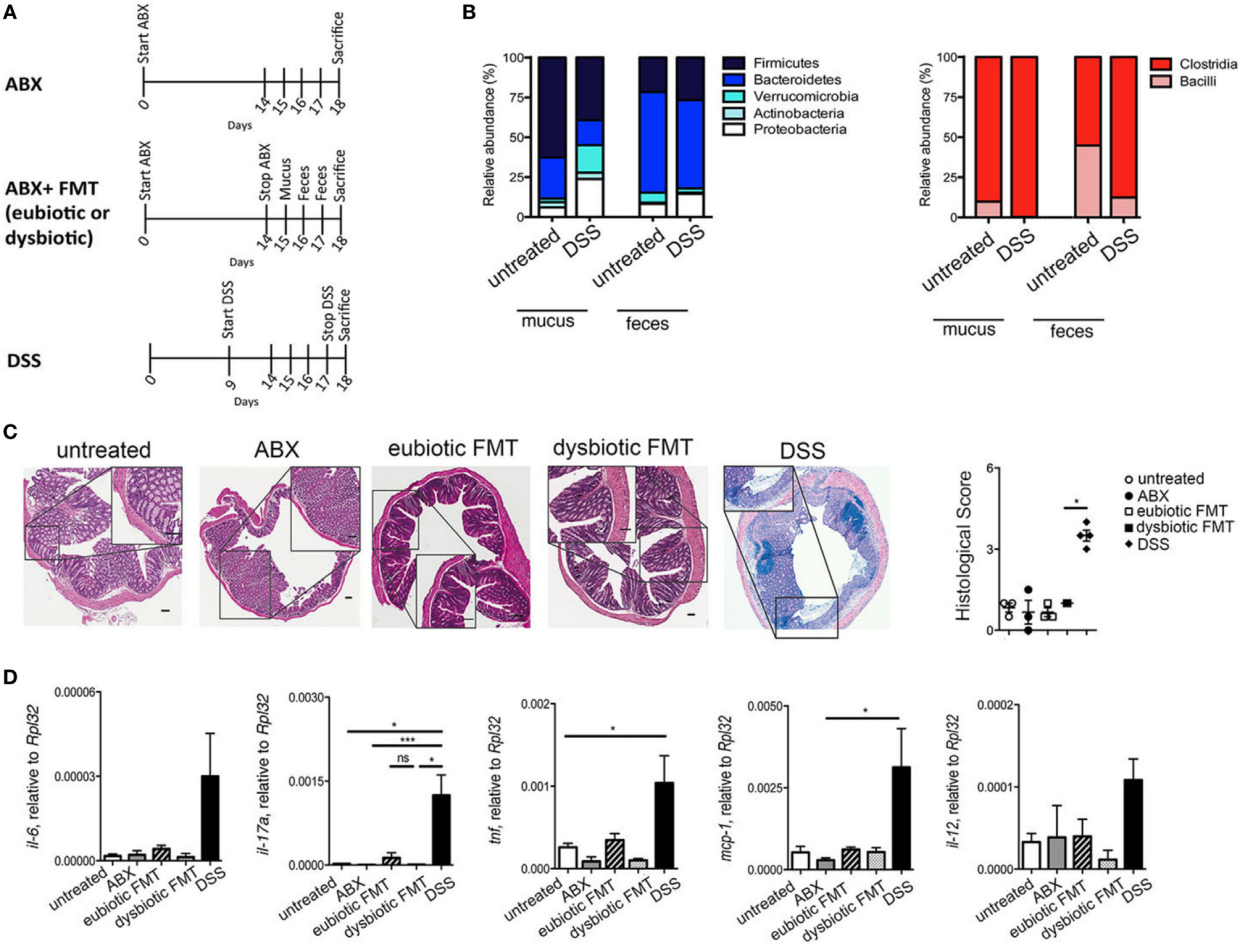
Antibiotic treatment does not alter mucosal architecture under homeostatic conditions. **(A)** Schematic representation of the treatments. **(B)** Abundances of dominant mucus-associated and fecal bacterial groups (phyla level on the left and class level among *Firmicutes* on the right) derived from untreated and DSS-treated mice and utilized to recolonize ABX-treated mice **(C)** H&E staining and cumulative histological score on colon specimens of untreated (open circles), antibiotic treated (ABX, closed circles), transplanted with eubiotic (open squares) or dysbiotic fecal microbiota transplantation (FMT) (closed squares), and of DSS-treated mice (closed diamonds). Scale bar 100 μm. **(D)** Colonic expression levels of *Il6, il17a, tnf, mcp-1, il-12* in untreated (white bars), ABX-treated (gray bars), reconstituted with eubiotic (striped bars) or dysbiotic (dotted bars) FMT or in DSS-treated mice (black bars). Significance was determined using Kruskal–Wallis nonparametric test and expressed as mean SEM untreated *n* = 8, ABX-treated *n* = 10, reconstituted with eubiotic FMT *n* = 9, with dysbiotic FMT *n* = 11, or in DSS-treated *n* = 11 mice in four independent experiments. Outliers detected with Grubb’s test. *P* < 0.05 (*), *P* < 0.001 (***) were regarded as statistically significant.

To assess the effects of antibiotic treatment on intestinal iNKT cells phenotype and function under homeostatic conditions, CXCR6-^EGFP/+^ mice were treated for 2 weeks with a broad-spectrum antibiotic cocktail (ABX, vancomycin, metronidazole, ampicillin, penicillin), targeting both aerobic and anaerobic bacteria (Figure 1A). CXCR6-^EGFP/+^ mice are a useful strain to track iNKT cells (24). The complete depletion of intestinal microbiota by ABX treatment was confirmed by colony forming unit analysis and qPCR analyses (data not shown). Subsequently, mice were either maintained in ABX treatment (ABX, upper scheme) or reconstituted by oral gavage with mucosa-associated and fecal bacteria (FMT; Figure 1A, middle scheme). To evaluate whether the characteristics of the microbiota utilized to reconstitute the gastrointestinal tract upon ABX treatment might have a subsequent impact on iNKT cells frequency and function, mucus and feces derived from healthy mice (eubiotic FMT) or from mice with intestinal dysbiosis (dysbiotic FMT) were engrafted to ABX-treated mice (Figure 1A, middle scheme). Dysbiotic microbiota was obtained from mice with acute DSS colitis, as we and others have observed that this condition is associated with a relevant alteration of both fecal and mucosa-associated microbial composition (25) (Figure 1B). Histological evaluation of colonic tissues (Figure 1C) and qPCR analyses of tissue derived mRNA (Figure 1D) revealed that neither antibiotic treatment nor recolonization with eubiotic or dysbiotic microorganisms induced macroscopic changes in the colonic tissue architecture (Figure 1C) and all treatments failed to upregulate inflammatory genes (Figure 1D).

**Figure 2:**
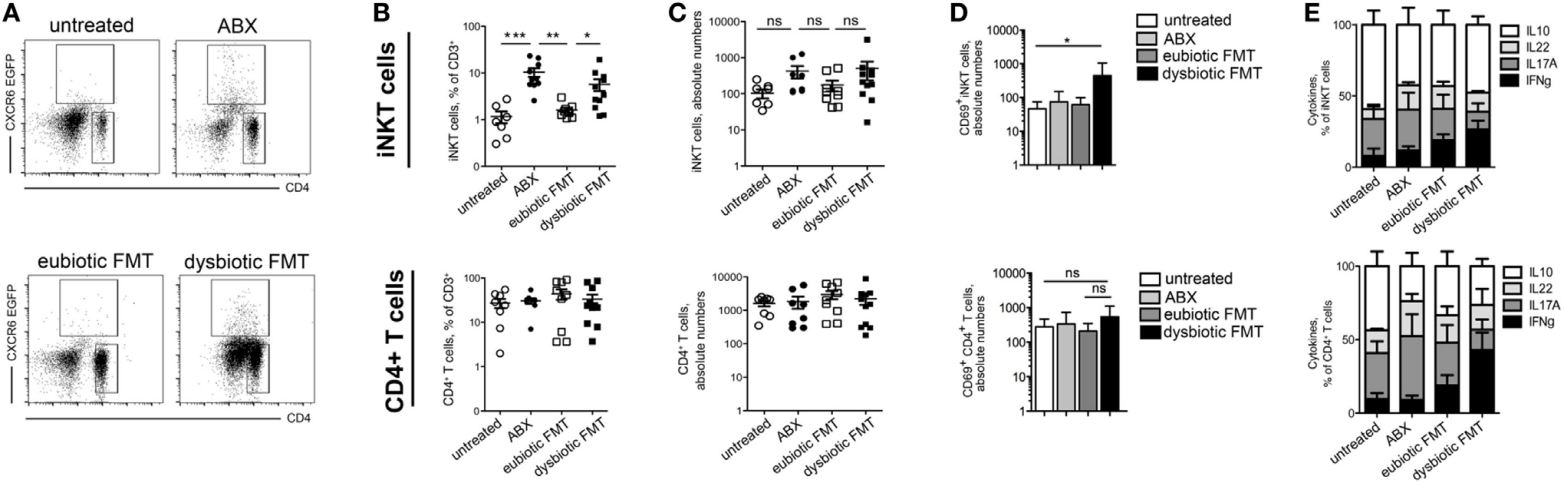
Antibiotic treatment influences colonic invariant natural killer T (iNKT) cell frequency and function **(A–C)** representative dot plots **(A)**, cumulative frequency **(B)**, and absolute numbers **(C)** of iNKT cells (upper panels) and CD4^+^ T cells (lower panels) in untreated mice (open circles), ABX-treated mice (closed circles), mice reconstituted with eubiotic fecal microbiota transplantation (FMT) (open squares), or with microbiota from DSS-treated mice (dysbiotic FMT, closed squares). **(D)** Absolute numbers of CD69^+^ cells among iNKT cells (upper panels) and CD4^+^ T cells (lower panels) in untreated mice (white bars), ABX-treated mice (light gray bars), mice reconstituted with eubiotic FMT (dark gray bars), or with microbiota from DSS-treated mice (dysbiotic FMT, black bars) **(E)** Cytokine production by iNKT cells (upper panels) and CD4^+^ T cells (lower panels) in untreated, ABX-treated, reconstituted with eubiotic or with dysbiotic FMT. Histograms normalized to 100% of production of total cytokines. Significance was determined using Kruskal–Wallis nonparametric test and expressed as mean SEM. Untreated *n* = 8, ABX-treated *n* = 10, reconstituted with eubiotic FMT *n* = 9, with dysbiotic FMT *n* = 11 or in DSS-treated *n* = 11 mice in four independent experiments. Outliers detected with Grubb’s test. *P* < 0.05 (*), *P* < 0.01 (**), *P* < 0.001 (***) were regarded as statistically significant.

In sharp contrast, treatment of adult mice with broad spectrum antibiotics was sufficient to induce a significant expansion of iNKT cells in the colon (Figures 2A–C; Figures S1 and S2 in Supplementary Material). Importantly, reconstitution of the gut microbiota with a eubiotic FMT restored iNKT cell frequency (Figures 2A–C), a phenomenon that was not observed upon microbial reconstitution with the microbiota derived from DSS-treated mice. In contrast with these data, CD4^+^ T cells accumulation in the colon was unaffected by antibiotic treatment or by microbiota recolonization, regardless its origin (Figures 2A–C).

The expression of CD69 (Figure 2D), a surface molecule associated to T cells functional activation, and the cytokine profile (Figure 2E) of both iNKT cells and CD4^+^ T cells isolated from ABX-treated mice did not significantly differ from that of cells isolated from mice reconstituted with an eubiotic microflora. Conversely, re-colonization of ABX-treated mice with a dysbiotic microbiota was sufficient to upregulate CD69 and to stimulate IFNg secretion by both iNKT cells (Figures 2D,E), and by CD4^+^ colonic T cells.

Interestingly, these effects were observed in iNKT cells isolated from the colon, but not from the mesenteric LNs (mLN) or from the spleens (Figure S3 in Supplementary Material) of treated mice, thus confirming that the tight regulation of iNKT cells frequency and functions operated by the commensal microbiota occurs in the colonic microenvironment rather than in the periphery (23).

Taken together, these results suggest that expansion of colonic iNKT cells and CD4^+^ T cells are differentially modulated upon antibiotic treatment in the absence of intestinal inflammation, and that the microbiota composition influences both the accumulation and the functional activation of colonic iNKT cells.

### Recolonization of Antibiotic-Treated Mice with a Dysbiotic Microbiota Aggravates Subsequent Intestinal Inflammation

**Figure 3:**
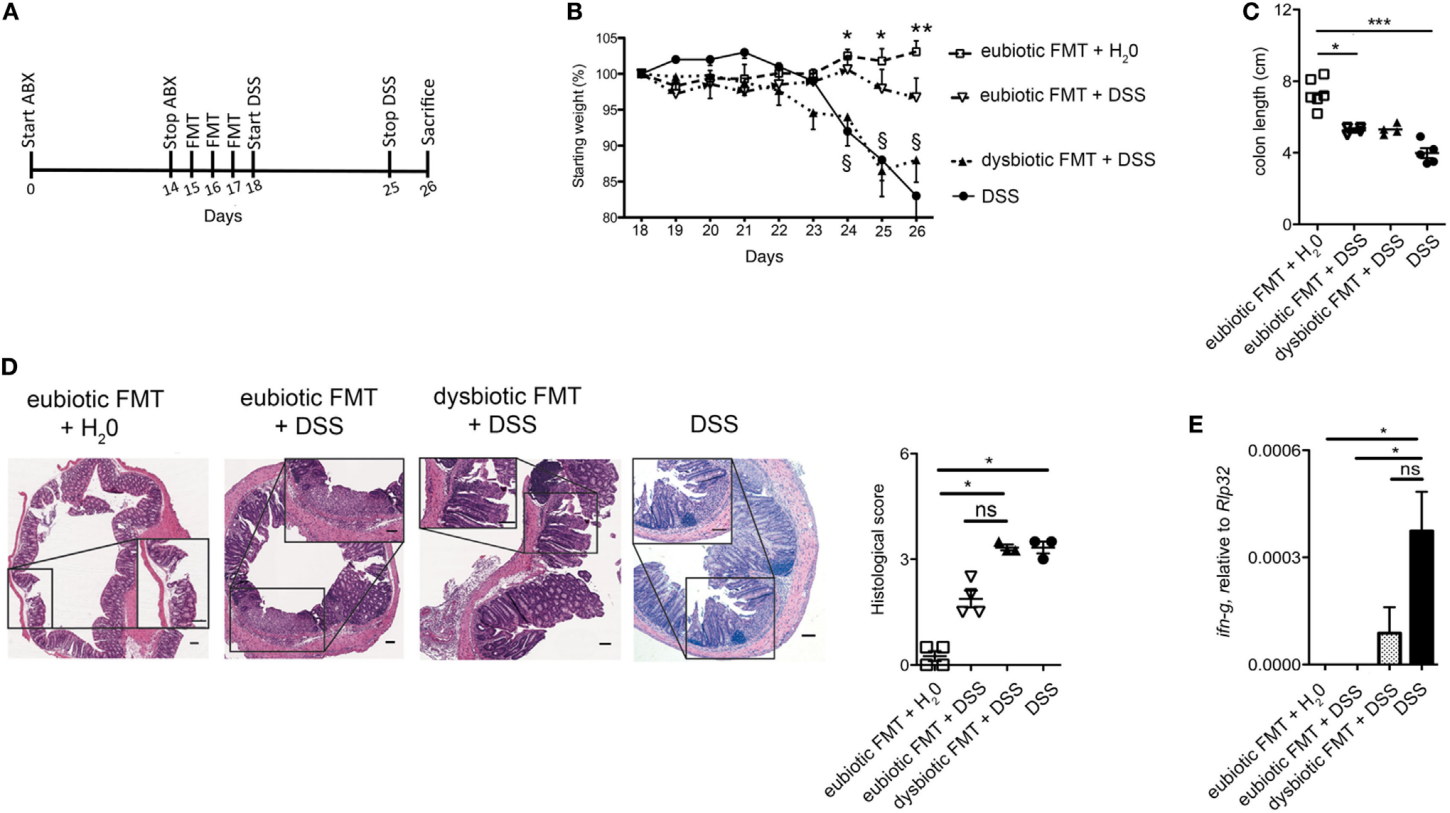
Reconstitution of ABX-treated mice with dysbiotic microbiota aggravates subsequent intestinal inflammation. **(A)** Schematic representation of the treatment. **(B)** Weight loss, **(C)** colon length, and **(D)** histological evaluation of mice treated with eubiotic FMT before water administration or treated with normal or dysbiotic fecal microbiota transplantation (FMT) before DSS administration, or treated with DSS without prior exposure to antibiotics. *Statistical analysis between normal FMT + H_2_0 and DSS; ^§^Statistical analysis between eubiotic FMT + H_2_0 and dysbiotic FMT + DSS performed with Mann–Whitney test. **(D)** Shows H&E staining on colon specimens. Scale bar 100 μm. **(E)** Colonic expression levels of *ifng* in mice reconstituted with eubiotic microbiota and unchallenged (gray bars), reconstituted with eubiotic bacteria and DSS-treated (striped bars), reconstituted with dysbiotic microbiota and DSS-treated (dotted bars) and DSS-treated (black bars). Eubiotic FMT + H_2_0 *n* = 6, eubiotic FMT + DSS *n* = 4, dysbiotic FMT + DSS *n* = 4, or DSS-treated *n* = 5 mice, two independent experiments. Significance was determined using Kruskal–Wallis nonparametric test and expressed as mean SEM Outliers detected with Grubb’s test. *P* < 0.05 (* and ^§^), *P* < 0.01 (** and ^§^), *P* < 0.001 (***) were regarded as statistically significant.

Since the nature of the microbiota utilized to reconstitute the gastrointestinal tract following antibiotic treatment exerted an influence over expansion and cytokine profile of colonic iNKT cells, we next assessed whether these effects translated into a parallel effect over experimental intestinal inflammation. To address this issue, mice were treated with broad-spectrum ABX for 2 weeks before reconstitution with either eubiotic or dysbiotic microbiota. Shortly after re-colonization, acute colitis was induced by administration of DSS in their drinking water (Figure 3A). Of note, mice recolonized with a dysbiotic microbiota exhibited a more severe colitis (as indicated by a more profound weight loss, Figure 3B, and a higher histological score, Figure 3D) than those reconstituted with a eubiotic microbiota. No difference was found in colon length (Figure 3C). Reconstitution with a dysbiotic microflora was sufficient to upregulate colonic *ifng* (Figure 3E).

Taken together, these results suggest that presence of an underlying dysbiotic microbiota may exert a negative influence over subsequent experimental intestinal inflammation.

### iNKT Cells Exposed to a Dysbiotic Microbiota Acquire an Activated and Pro-inflammatory Phenotype

**Figure 4:**
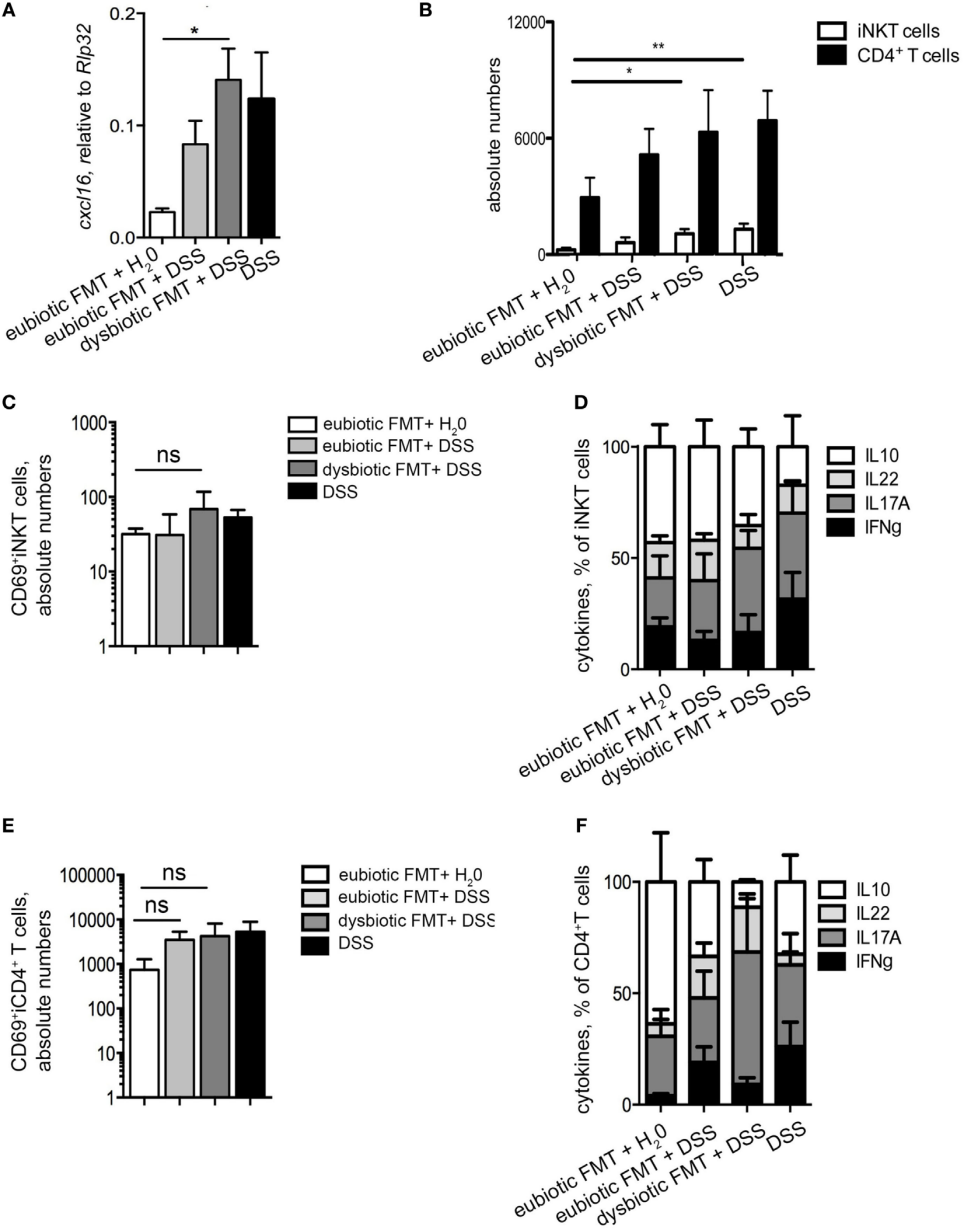
T cell responses are affected by the origin of the transplanted microbiota. **(A)** Colonic expression levels of *cxcl16* in mice reconstituted with eubiotic bacteria and unchallenged (white bars), reconstituted with eubiotic bacteria and DSS-treated (light gray bars), reconstituted with dysbiotic bacteria and DSS-treated (dark gray bars) and DSS-treated (black bars). **(B)** Absolute numbers of colonic invariant natural killer T (iNKT) cells (white bars) and CD4^+^ T cells (black bars) in the indicated experimental groups. **(C,E)** CD69 absolute numbers of colonic iNKT cells **(C)** and of colonic CD4^+^ T cells **(E)**. **(D,F)** Cytokine production by iNKT cells **(D)** and of CD4^+^ T cells **(F)** in the indicated experimental groups. Histograms normalized to 100% of production of total cytokines. Significance was determined by Kruskal–Wallis nonparametric test. Eubiotic fecal microbiota transplantation (FMT) + H_2_0 *n* = 6, eubiotic FMT + DSS *n* = 4, dysbiotic FMT + DSS *n* = 4, or DSS-treated *n* = 5 mice, two independent experiments. Outliers detected with Grubb’s test. *P* < 0.05 (*), *P* < 0.01 (**) were regarded as statistically significant.

We next evaluated if the observed negative effects of the re-colonization with a dysbiotic microbiota after antibiotic treatment were associated to a specific phenotype of colonic iNKT cells. First, the colonic expression levels of *cxcl16*, the chemokine responsible for iNKT cells tissue attraction (22), were evaluated (Figure 4A). Colonic *cxcl16* levels were strongly upregulated by DSS treatments, as compared to its level after reconstitution with eubiotic FMT in the absence of inflammation (Figure 4A). Noteworthy, reconstitution of ABX-treated mice with dysbiotic microbiota-induced *cxcl16* expression at similar levels as those of DSS without prior ABX treatement. Consistently, a marked accumulation of iNKT cells, and also of CD4^+^ T cells, was observed in the colon of mice upon DSS administration (Figure 4B).

As a consequence of higher cxcl16 expression and increased chemoattraction, colonic iNKT cells were more abundant in mice reconstituted with a dysbiotic microbiota than those reconstituted with a eubiotic microbiota (Figure 4B).

Additionally, a tendency of a higher accumulation of colonic iNKT cells expressing CD69 was observed in colitic mice re-colonized with a dysbiotic microbiota after ABX treatment (Figure 4C). Similarly to CD69 expression, the cytokine profile of colonic iNKT cells isolated from colitic mice re-colonized with dysbiotic microbiota was skewed toward a pro-inflammatory Th1/Th17 cytokine profile (Figure 4D), which in CD4^+^ T cells is associated to pathogenic properties (26). On the contrary, re-colonization of the gastrointestinal tract with a eubiotic microbiota did not sustain the activated/inflammatory phenotype of iNKT cells, but rather maintained them toward an uninflamed/IL10-secreting cytokine profile (Figure 4D).

As for colonic CD4^+^ T cells, no differences were observed among DSS-treated groups in terms of CD69 expression (Figure 4E), although also CD4^+^ T cells isolated from mice re-colonized with dysbiotic microbiota before colitis induction were similarly skewed toward a Th1/Th17 cytokine profile (Figure 4F).

Taken together, these data indicate that re-colonization of the gastrointestinal tract after antibiotic treatment with a dysbiotic microbiota, but not with an eubiotic microbiota, sustains the accumulation of iNKT cells (and of CD4^+^ T cells) with an activated and pro-inflammatory/colitogenic phenotype.

### Dysbiotic Microbiota Effects after Antibiotic Treatment on Colonic iNKT Cells Are Time-Dependent

**Figure 5:**
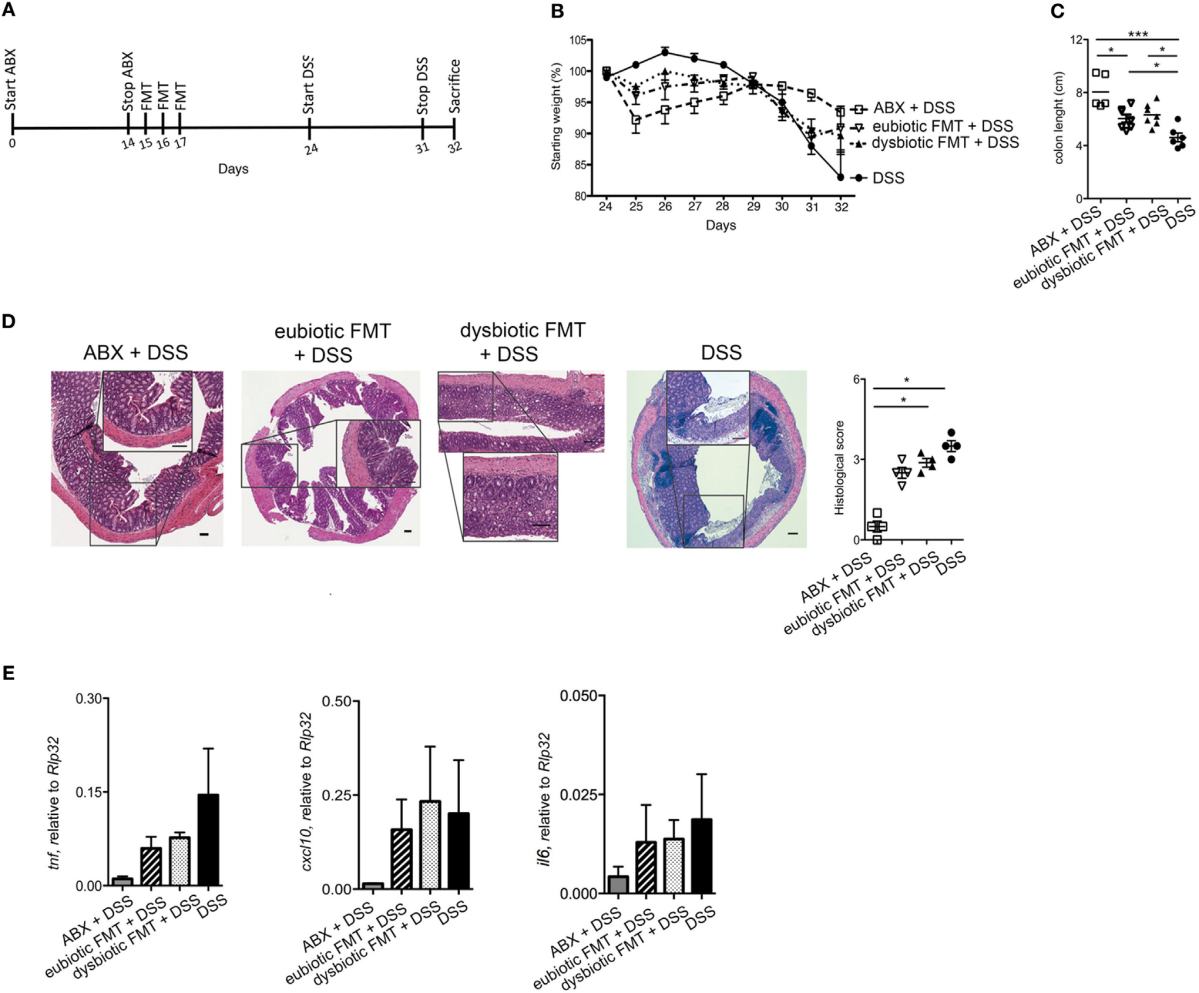
Dysbiotic fecal microbiota transplantation (FMT) effects are time-dependent **(A)** Schematic representation of treatment. **(B)** Weight loss, **(C)** colon length, and **(D)** histological evaluation of mice treated with ABX before DSS administration or treated with eubiotic or dysbiotic FMT before DSS administration or with DSS. **(D)** Shows H&E staining on colon specimens. Scale bar 100 μm. **(E)** Colonic expression levels of *tnf, cxl10, and il6* in ABX-treated mice before DSS treatment (gray bars), reconstituted with eubiotic bacteria and DSS-treated (striped bars), reconstituted with dysbiotic bacteria and DSS-treated (dotted bars) and DSS-treated (black bars). Significance determined using Kruskal–Wallis nonparametric test and expressed as mean SEM *P* < 0.05 (*), *P* < 0.01 (**), *P* < 0.001 (***) were regarded as statistically significant.

We next aimed to evaluate whether the negative effects of re-colonization with dysbiotic microbiota over intestinal inflammation were persistent over time (Figure 5A). To this end, mice were treated with ABX for 2 weeks and then reconstituted with either a eubiotic or a dysbiotic microbiota. This time, DSS administration was performed 1 week after the last microbiota gavage (Figure 5A). By following this protocol, no differences could be observed between the effects of eubiotic and dysbiotic re-colonization after antibiotic treatment in terms of weight loss (Figure 5B), colon length reduction (Figure 5C), histological score (Figure 5D), and expression of inflammatory genes in the colonic tissue (Figure 5E).

**Figure 6:**
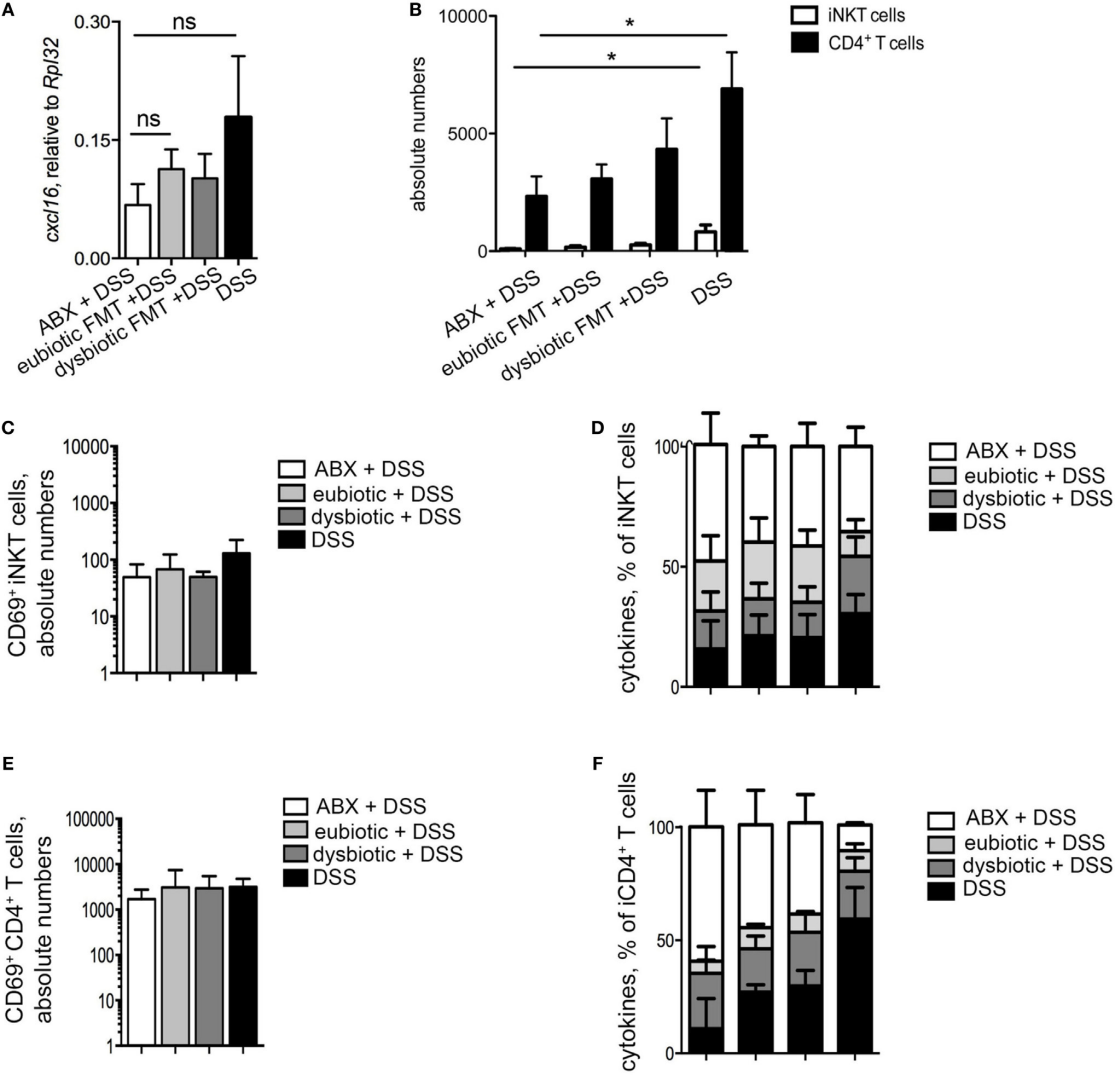
Dysbiotic microbiota effects after antibiotic treatment on colonic invariant natural killer T (iNKT) cells are time-dependent. **(A)** Colonic expression levels of *cxcl16* in mice treated with antibiotics and treated with DSS (white bars), transplanted with eubiotic bacteria and DSS-treated (light gray bars), transplanted with dysbiotic bacteria, and DSS-treated (dark gray bars) and DSS-treated (black bars). **(B)** Absolute numbers of colonic iNKT cells (white bars) and CD4^+^ T cells (black bars) in the indicated experimental groups. **(C,E)** CD69 absolute numbers of colonic iNKT cells **(C)** and of colonic CD4^+^ T cells **(E)**. **(D,F)** Cytokine production by iNKT cells **(D)** and CD4^+^ T cells **(F)** in the indicated experimental groups. Histograms normalized to 100% of production of total cytokines. Significance determined using Kruskal–Wallis nonparametric test and expressed as mean SEM. ABX + DSS *n* = 5, eubiotic FMT + DSS *n* = 6, dysbiotic FMT + DSS *n* = 7, DSS-treated *n* = 6 mice, two independent experiments. Outliers detected with Grubb’s test. *P* < 0.05 (*) were regarded as statistically significant.

Similarly, the difference in colonic *cxcl16* expression between mice re-colonized with eubiotic or dysbiotic microbiota was abolished when a longer time was allowed before colitis induction (Figure 6A), mirrored by similar recruitment and abundance of colonic iNKT cells among the two groups (Figure 6B). A similar re-equilibration between the effects of eubiotic and dysbiotic reconstitution before colitis induction was observed also on the activation status (Figure 6C) and cytokine profile (Figure 6D) of iNKT cells. To note, the differences of pro-inflammatory phenotype induction on conventional CD4^+^ T cells between eubiotic and dysbiotic microbiota exposure were also abolished (Figures 6E,F).

Taken together, these results confirm that the pro-inflammatory effects of the dysbiotic microbiota over colitis development might be significantly affected by the time allowed for microbial reconstitution.

## Discussion

The immune system and the host microbiota shape each other throughout life, generating an equilibrium that is daily challenged as a result of exposure to pathogens, to environmental factors, or to dietary changes (1). Recently, antibiotic usage has been identified as one important trigger of disequilibrium between the immune system and the intestinal microbiota (8), with still poorly understood consequences on the overall human health.

We here report that a short-term broad-spectrum antibiotic administration is sufficient to affect the phenotype and function of colonic iNKT cells and that the re-colonization of the intestinal microbiota with dysbiotic, but not with eubiotic, microorganisms aggravates subsequent intestinal inflammation by sustaining T cells pro-inflammatory phenotype.

A healthy microbial ecosystem is defined by its richness and its resistance to external perturbations. Data from the Human Microbiome Project (27) indicate that multiple eubiotic states of the microbial ecosystem, whose taxonomic compositions are influenced by geography, age, and dietary habits, co-exist throughout life even without manifest signs of disease (7). Dysbiosis can be defined as any short-term or stable alteration in the microbial ecology affecting the taxonomical composition as well as the metagenomic functions of the microbial community (7). Growing evidences support the notion that antibiotic exposure cause short-term and long-term intestinal dysbiosis by reducing or completely removing normally residing members of the microbiota, as a consequence of microbial killing or diminished bacterial proliferation (8, 28, 29).

Even though it has been extensively shown that microbiota absence in GF mice impacts the immune system development and function (3, 30), the effects of antibiotic-dependent microbiota ablation on the mucosal immune system has so far been poorly studied. Antibiotic exposure in early life reduces bacterial diversity and alters the composition of the commensal microbiota (8), a phenomenon linked to increased susceptibility to develop immune-related pathologies in adult life such as allergic inflammation and asthma (10, 11).

A recent report indicate that prolonged (8 weeks) broad spectrum ABX usage (16) induces a reduction in the relative abundance of several immune cell populations, including T cells, both in periphery and in the intestinal tissues. This event can be reverted by re-colonization of antibiotic-treated mice with a normal microflora.

Here, we show that also a shorter exposure to a broad-spectrum ABX cocktail, a schedule of treatment closer to the ones used in clinical practice is sufficient to induce alterations in the relative abundances and cytokine profile of lipid-specific iNKT cells and of conventional CD4^+^ T cells. Interestingly, 2 weeks ABX administration does not induce macroscopic signs of inflammation, as demonstrated by unaltered colonic architecture and low expression of inflammatory genes by the colonic mucosa, but it is sufficient to expand colonic iNKT cells and imprint them toward an inflammatory profile.

We observed that upon antibiotic treatment, only colonic iNKT cells, but not those residing in the mLN or in the spleen, increased in frequency. Alterations in the survival of colonic iNKT cells or the incremented availability of colonic CXCL16 might be associated to iNKT cells frequency increase upon antibiotic treatment. In addition, this observation can be explained by the notion that iNKT cell proliferative capacity is negatively regulated by the presence in the intestinal microflora of the commensal microbe *B. fragilis* (22). In GF mice, where *B. fragilis* is absent, mucosal iNKT cells expand without control (22). Similarly, broad-spectrum antibiotics induce a rapid and significant drop in taxonomic richness and diversity, including a reduction in Bacteroidetes (28, 29), thus suggesting a similar explanation for the observed iNKT cell frequency increase in ABX-treated mice. Re-colonization of the gastrointestinal tract with a Bacteroidetes-rich healthy microbiota explains the observed iNKT cells frequency normalization upon eubiotic bacterial reconstitution.

Colonic conventional CD4^+^ T cells are also influenced by the presence or absence of specific bacterial strains (4, 5). GF mice manifest impairments in CD4^+^ T cell functions (1, 30) while Vancomycin administration, selectively ablating Gram-negative bacteria, causes reduction of Tregs in colonic LP (5, 31). In our model, we did not observe variations in the frequency of CD4^+^ T helper (Th) cells, neither in the colon nor in the mesenteric LN or spleen, after 2 weeks broad-spectrum antibiotic administration. On the contrary, it was previously shown that a longer ABX treatment (8 weeks) could affect CD4^+^ and CD8^+^ T cells intestinal frequencies (16), suggesting that a shorter antibiotic exposure may not be sufficient to induce changes in the expansion of the MHC-restricted T cell population, as instead observed for non-classical lipid-specific iNKT cells.

We also observed that ABX treatment induced a skewing toward a pro-inflammatory cytokine profile in both colonic iNKT cells and conventional CD4^+^ T cells. No data were available so far on cytokine profile skewing upon short-term antibiotic treatment in colonic T cells. It was reported that a cocktail of ABX for 2 weeks was capable to skew the cytokine profile of systemic T cells promoting Th2 responses (32), similarly to short-term Kanamycin administration in 3-week-old mice (33). A longer antibiotic treatment, instead, completely abrogated the overall cytokine production by intestinal CD4^+^ T cells (16), supporting the hypothesis that the duration of antibiotic treatment might differentially impact on the cytokine producing capability of intestinal T cells.

It has also been shown that re-association of antibiotic-treated mice with distinct bacterial species could alter the cytokine profile of conventional T cells (33). *Enterococcus faecalis* and *Lactobacillus acidophilus* could revert or attenuate the cytokine skewing of conventional Th subsets, while *Bacteroides vulgatus* caused exacerbation of Th-2 inflammatory responses (33).

Intestinal inflammation induces profound alterations of the gut microbiota ecosystem (34). IBD patients harbor an important bacterial diversity as compared to not-IBD controls, defined by an increase in Proteobacteria (such as *E. coli adherent invasive* and Enterobacteriaceae in CD) and a decrease in Firmicutes (such as *F. prausnizii*) (35). Also, DSS-treated mice harbor a dysbiotic microbiota enriched in pathobionts (25), namely commensal microorganisms that bear the potential to cause pathology. These types of microorganisms are enriched in human IBD (36) and in murine models of intestinal inflammation (25, 37) and when transferred into GF are sufficient to induce experimental intestinal inflammation (4, 37).

In the context of antibiotic-premedicated mice, we observed that re-colonization of the gastrointestinal tract with dysbiotic microbiota, but not with eubiotic microbiota, directly affected the activation status of iNKT cells (and of conventional CD4^+^ T cells), as demonstrated by their upregulation of CD69 and their skew toward a Th1/Th17 pro-inflammatory cytokine phenotype. At present, it remains to be elucidated whether iNKT cell activation depends on the recognition of stimulatory lipid antigens or rather on innate signals originated from bacterial pattern recognition molecules.

In this work, we observed that the functional imprinting of iNKT cells and of CD4^+^ T cells by a dysbiotic microbiota toward an activated/inflammatory Th1-Th17 cytokine profile after antibiotic treatment had important consequences when mice experienced subsequent intestinal inflammation. The transferred dysbiotic microbiota utilized to re-colonize mice showed a higher colitogenic potential than eubiotic microbiota in mice premedicated with antibiotics, and both iNKT cells and CD4^+^ T cells manifested a sustained Th1/Th17 skewed cytokine profile in this experimental condition. Indeed, in line with previous reports (38), this observation suggests that antibiotic treatment might alter the colonization niche, favoring the selective engraftment of pathogenic bacteria.

Induction of a pro-inflammatory phenotype by colonic T cells after antibiotic treatment by a dysbiotic microbiota might potentially have clinically relevant consequences. Patients that are genetically susceptible to harbor a dysbiotic microbiota, like those suffering from IBD, during the course of their disease might undergo treatments with antibiotics and experience episodes of intestinal inflammation. Indeed, current guidelines of ECCO (39, 40) do not recommend antibiotic treatment for IBD patients, unless infective complications are suspected or ongoing and before surgical interventions.

As observed in our experimental model, upon cessation of antibiotic treatment, IBD patients could re-colonize their gastrointestinal tract with a dysbiosis-prone microbiota, capable to induce Th1/Th17 secreting colonic (iNK)T cells and bearing an activated phenotype. Thus, the synergistic action of antibiotics and the dysbiotic microbiota might potentially aggravate the outcomes of IBD-related inflammation.

Our experimental data suggest that, at least in the absence of genetically predisposing factors, a longer time between microbiota engraftment and DSS-induced intestinal inflammation is sufficient to reduce the colitogenic potential of the dysbiotic microbiota. If this effect is secondary to the filling of the colonization niche by normal eubiotic bacteria remains to be elucidated. Nonetheless, it opens the possibility to evaluate therapeutic interventions, for example, by administering probiotics, to contrast the engraftment of pathobionts after antibiotic treatment.

## Summary and Conclusion

In this study, we observed that antibiotic treatment profoundly altered the frequency of intestinal iNKT cells and the functions of both iNKT and CD4^+^ T cells even in the absence of concomitant intestinal inflammation. The presence of a dysbiotic microbiota after antibiotic treatment imprints colonic (iNK)T cells toward a pro-inflammatory phenotype that contributes to aggravate intestinal inflammation. Nonetheless, we observed that the inflammatory potential of the dysbiotic microbiota decreased over time, at least in a system uncoupled with genetic predisposition to select and maintain the engraftment of pathobionts. At present, it remains to be elucidated whether (iNK)T cells activation in these conditions depends on the recognition of stimulatory lipidic or proteic antigens or rather on innate signals conveyed by the dysbiotic microbiota.

In immune-mediated intestinal pathologies, it has not been fully elucidated whether intestinal dysbiosis may be the cause or a consequence of intestinal inflammation. However, transfer of dysbiotic microflora or single commensal bacterial species in GF is sufficient to induce experimental intestinal inflammation and activate the mucosal immune system (4, 37). What it is now emerging is that antibiotic treatment can also induce dysbiosis and, as we observed, this can promote activating pro-inflammatory immune cell responses in the colon.

Individuals with genetic predispositions to harbor a dysbiotic microbiota and to aberrantly activate the mucosal immune system are, therefore, more exposed to unwanted pro-inflammatory immune responses after antibiotic treatment.

Albeit antibiotic treatment cannot be avoided, it should be kept in mind to predispose, especially for those individuals, treatments to re-equilibrate antibiotic-induced dysbiosis. For example, probiotics administration could contribute to contrast pathobionts engraftment and simultaneously switching-off mucosal immune cell activations.

## Ethics Statement

This study was carried out in accordance with the recommendations of the European Guideline for animal welfare (2010/63/EU). Animal procedures were approved by the Italian Ministry of Health (Auth. 127/15, 27/13, 913/16).

## Author Contributions

CB performed experiments, analyzed and interpreted data. FG performed experiments and analyzed data. FMC and GE performed and interpreted histological analyses. SB critically revised the manuscript. FC contributed to interpretation of the data and gave important intellectual contributions and revised the manuscript. FF designed, conceived, and supervised the study and wrote the manuscript.

## Conflict of Interest Statement

The authors declare that the research was conducted in the absence of any commercial or financial relationships that could be construed as a potential conflict of interest.

## Abbreviations

iNKT, invariant natural killer T cells; GF, germ free; ABX, antibiotics; Th, T helper; IBD, inflammatory bowel diseases; FMT, fecal microbiota transplantation.
