# Supplementary material for: Short-term Oral Antibiotics Treatment Promotes Inflammatory Activation of Colonic Invariant Natural Killer T and Conventional CD4+ T Cells
Source: Front Med (Lausanne). 2018 Feb 7;5:21. doi: 10.3389/fmed.2018.00021 (PMC5808298; doi:10.3389/fmed.2018.00021)
Supplement: Supplementary file 1 [file Data_Sheet_1.docx]

Supplementary Material

**Short-term oral antibiotics treatment promotes inflammatory activation of colonic iNKT and conventional CD4^+^ T cells**

Claudia Burrello, Federica Garavaglia, Fulvia Milena Cribiù, Giulia Ercoli, Silvano Bosari, Flavio Caprioli, Federica Facciotti^*^

- Correspondence: Corresponding Author: [Federica.facciotti@ieo.it](mailto:Federica.facciotti@ieo.it)

**1. Supplementary Figures and Tables**

## Supplementary Figures

##
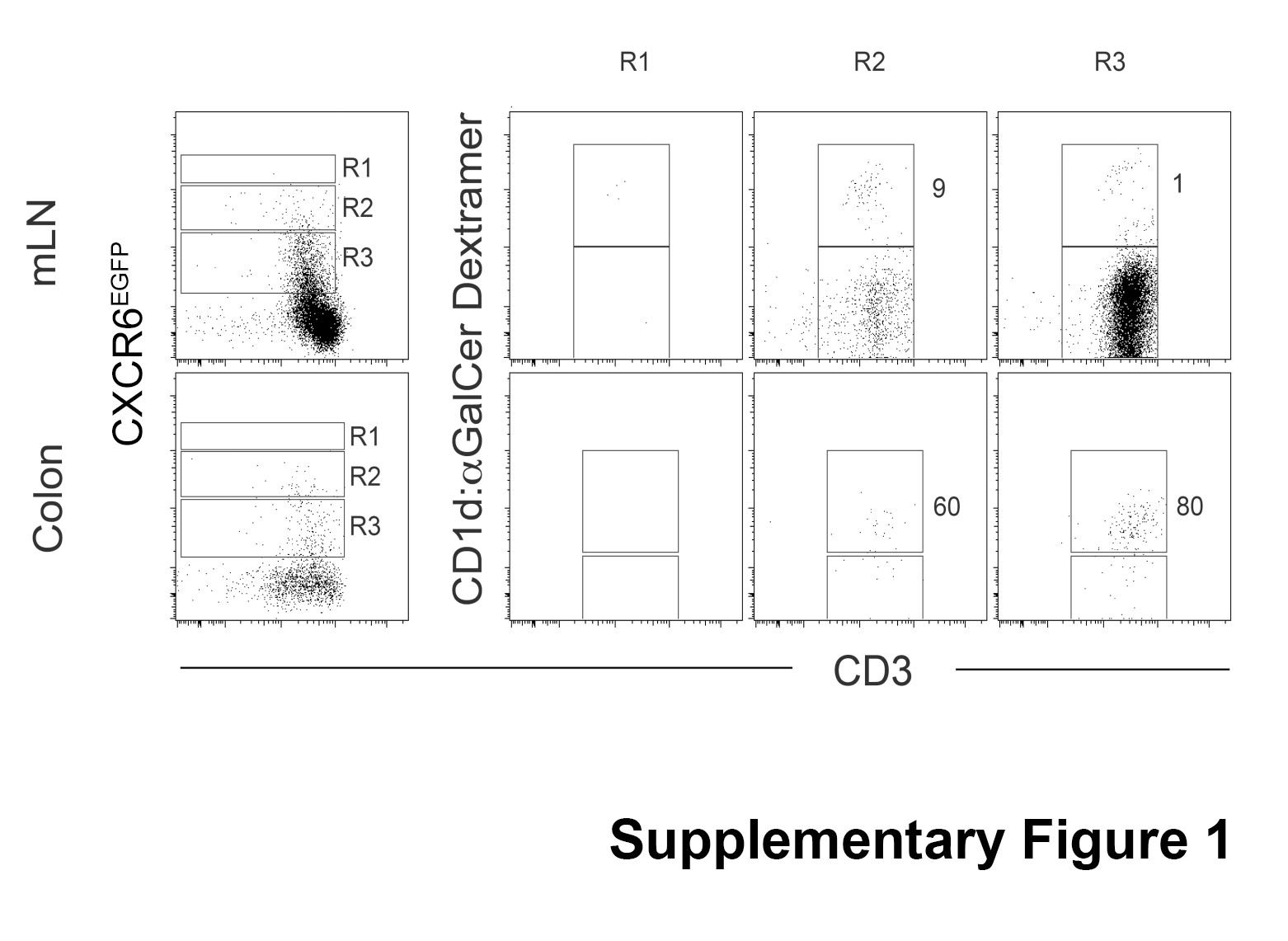


**Supplementary Figure 1.** CXCR6^EGFP^ expression in mesenteric LN (upper panel) and colon (lower panel) as compared to mCD1d:PBS57 Tet staining on gated Lin negCD3^+^ cells

**
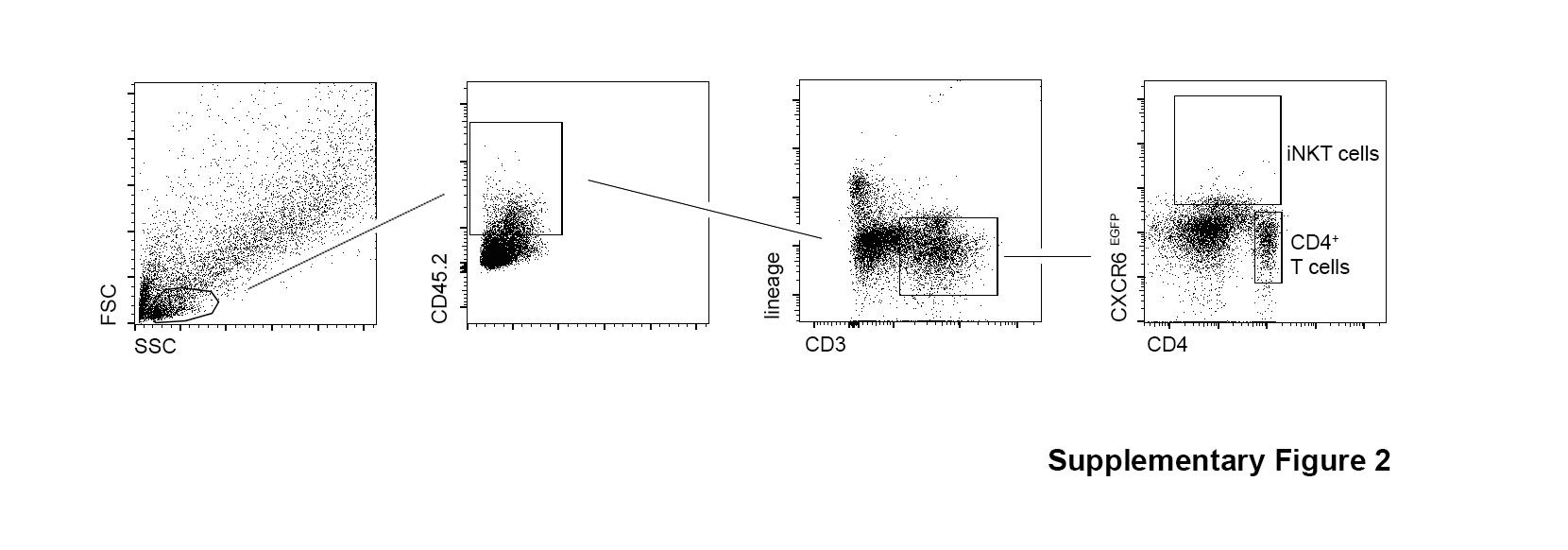
**

**Supplementary Figure 2:** *Gating strategy to identify iNKT cells in CXCR6 ^EGFP/+^ mice.* (A) Forward and Side Scatter of colonic LPMC gate indicates living lymphocytes (B) CD45.2 gate to exclude epithelial cells. (C) Gate to exclude lineage^+^ (CD19, CD11c, CD11b) cells and select CD3+ cells (D) CD4 expression and CXCR6-^EGFP^


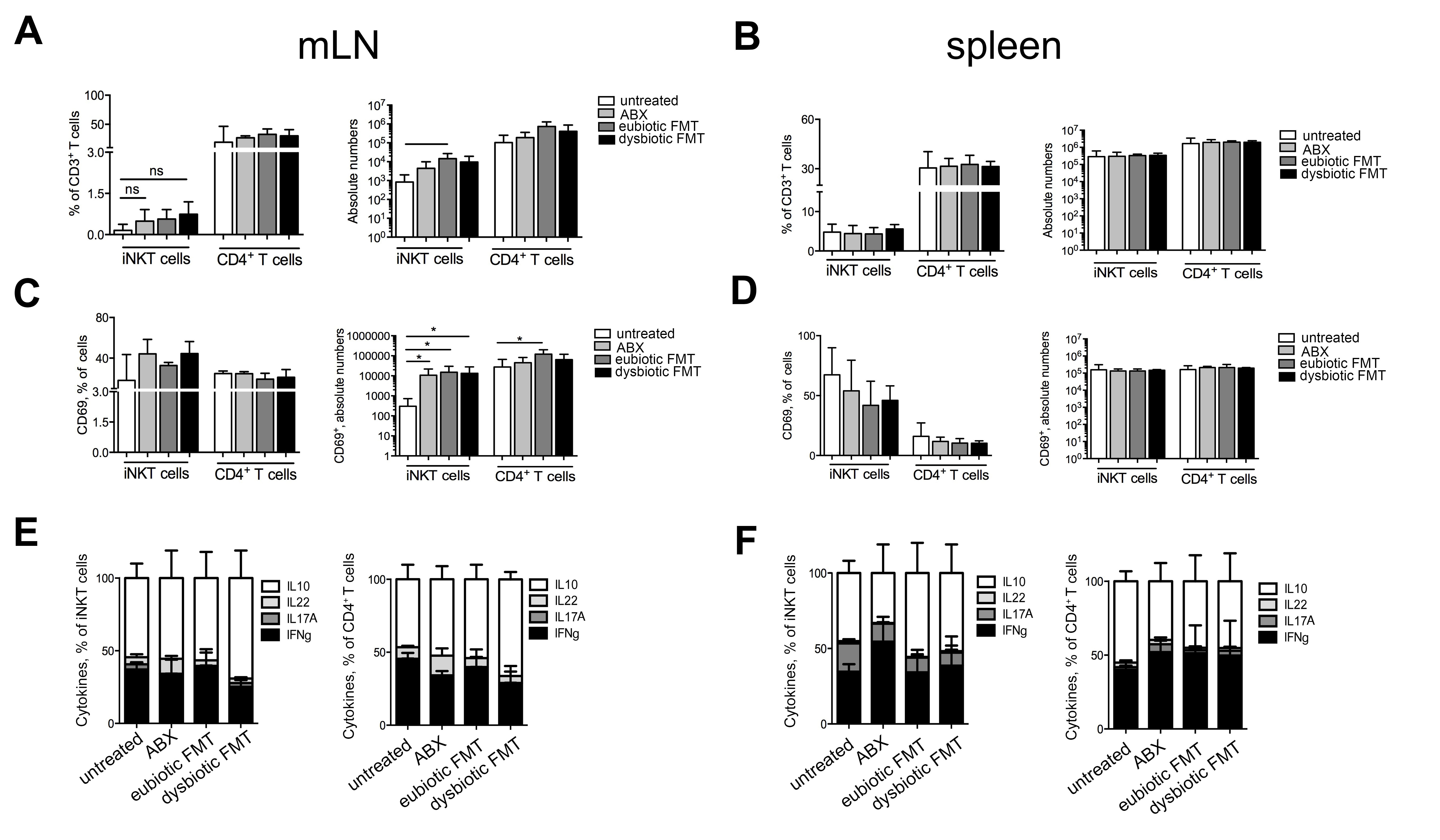


**Supplementary Figure 3**: *Antibiotic treatment does not influences iNKT cell frequency and function in mLN and spleen (*A-B) cumulative frequency and absolute numbers of iNKT cells and CD4+ T cells in untreated mice (white bars), ABX-treated mice (light gray bars), mice reconstituted with eubiotic FMT (dark gray bars) or with microbiota from DSS-treated mice (dysbiotic FMT, black bars) in (A) mLN and (B) spleen. (C, D) Absolute numbers of CD69+ cells among iNKT cells and CD4+ T cells (lower panels) in untreated mice (white bars), ABX-treated mice (light gray bars), mice reconstituted with eubiotic FMT (dark gray bars) or with microbiota from DSS-treated mice (dysbiotic FMT, black bars) in (C) mLN and (D) spleen. (E,F) Cytokine production by iNKT cells (left panels) and CD4^+^ T cells (right panels) in untreated, ABX-treated, reconstituted with eubiotic or with dysbiotic FMT in (E) mLN and (F) spleen. Histograms normalised to 100% of production of total cytokines. Significance was determined using unpaired two-tailed Student’s t test and expressed as mean SEM. Untreated n=8, ABX-treated n=10, reconstituted with eubiotic FMT n=9, with dysbiotic FMT n=11 or in DSS-treated n=11 mice in 4 independent experiments. Outliers detected with Grubb’s test. P < 0.05 (*), P < 0.01 (**) P < 0.001 (***) were regarded as statistically significant. iNKT cells from mLN were detected by CD1d:PBS57 Tetramer staining (NIH Tetramer facility)

## Supplementary Tables

**Table 1: Primer sequences**

| **Primer** | **Product size (bp)** | | | **Vendor** |
| --- | --- | --- | --- | --- |
| IL17 | 94 | | | Qiagen (QuantiTect) |
| IFNg | 190 | | | Qiagen (QuantiTect) |
| IL10 | 103 | | | Qiagen (QuantiTect) |
| IL23 | 80 | | | Qiagen (QuantiTect) |
| Rpl32 | 117 | | | Qiagen (QuantiTect) |
|  |  | | |  |
| **Primer** | **Forward** | **Reverse** | **bp** | **Vendor** |
| CXCL16 | AGCGCAAAGAGTGTGGA | GGTTGGGTGTGCTCT | 193 | SIGMA |
| MCP-1 | CAAGATGATCCCAATGA | GGTTCCGATCCAGGT | 161 | SIGMA |
| CXCL10 | CGCTGCAACTGCCATCCA | CCGGATTCAGACATC | 148 | SIGMA |
| TNF | TCTTCTCATTCCTGCTTG | CACTTGGTGGTTTGCT | 200 | SIGMA |
| IL6 | CTCTGGGAAATCGTGGA | GCAAGTGCATCATCG | 77 | SIGMA |
| IL12 | CCTGCTGAAGACCACAG | AGCTCCCTCTTGTTGT | 200 | SIGMA |
|  |  |  |  |  |
| **PCR assay** | **Forward 5’-> 3’** | **Reverse 5’-> 3’** | **Target gene** | **Vendor** |
| *Acetivibrio spp.* | GAAGATGTAATGATAGTTGC | GTCTATTGCATTTGGAACTGA | cipV | SIGMA |
| *A.muciniphila* | CAGCACGTGAAGGTGGGGAC | CCTTGCGGTTGGCTTCAGAT | 16S rRNA | SIGMA |
| *Bacillus spp.* | CCAGTAGCCAAGAATGGCCAGC | GGAATAATCGCCGCTTTGTGC | eglS3 | SIGMA |
| *Bacteroides spp.* | GGARCATGTGGTTTAATTCGATGAT | AGCTGACGACAACCATGCAG | 16S rRNA | SIGMA |
| *Bifidobacterium spp.* | GCGTGCTTAACACATGCAAGTC | CACCCGTTTCCAGGAGCTATT | 16S rRNA | SIGMA |
| *Blautia spp.* | CGGTATGTAAACTTCTATCAGCA | CAGTTTCCAATGCAGTCC | 16S rRNA | SIGMA |
| *Butyruvibrio spp.* | GTGCCAGCMGCCGCGG | TGCGGCACYGACTCCCTATG | 16S rRNA | SIGMA |
| *C.arthromitus (SFB)* | GACGCTGAGGCATGAGAGCAT | GACGGCACGGATTGTTATTCA | 16s rRNA | SIGMA |
| *Clostridium spp.* | GCACAAGCAGTGGAGT | CTTCCTCCGTTTTGTCAA | 16s rRNA | SIGMA |
| *Desulfovibrio spp.* | CCGTAGATATCTGGAGGAACATCAG | ACATCTAGCATCCATCGTTTACAGC | 16s rRNA | SIGMA |
| *E.coli* | CATGCCGCGTGTATGAAGAA | CGGGTAACGTCAATGAGCAAA | 16s rRNA | SIGMA |
| *Enterococcus spp.* | CCCTTATTGTTAGTTGCCATCATT | ACTCGTTGTACTTCCCATTGT | 16s rRNA | SIGMA |
| *Lactobacillus spp.* | AGCAGTAGGGAATCTTCCA | CACCGCTACACATGGAG | 16s rRNA | SIGMA |
| *Parabacteroides spp.* | TGATCCCTTGTGCTGCT | ATCCCCCTCATTCGGA | 16S-23S rDNA | SIGMA |
| *Prevotella spp.* | CACRGTAAACGATGGATGCC | GGTCGGGTTGCAGACC | 16s rRNA | SIGMA |
| *Propionibacterium spp.* | AGTGGCGAAGGCGGTTCTCTGGA | TGGGGTCGAGTTGCAGACCCCAAT | 16s rRNA | SIGMA |
| *Pseudomonas spp.* | CTACGGGAGGCAGCAGTGG | TCGGTAACGTCAAAACAGCAAAGT | 16s rRNA | SIGMA |
| *Ruminococcus spp.* | CCTCTGACCGCTCTTTAATCGGAGCTTTCCTTC | CCAGTTATCGGTCCCACCTTCGGCAGCT | 16s rRNA | SIGMA |
| *S. thyphimurium* | CATTGACGTTACCCGCAGAAGAAGC | CTCTACGAGACTCAAGCTTGC | 16s rRNA | SIGMA |
| *Streptococcus spp.* | GTACAGTTGCTTCAGGACGTATC | ACGTTCGATTTCATCACGTTG | tuf | SIGMA |

**Table 2: Scoring scheme for the evaluation of intestinal inflammation**

| **Category** | **Criterion** | **Definition** | **Score value** | |
| --- | --- | --- | --- | --- |
| **Inflammatory cell infiltrate** | Severity (leukocyte density of lamina propria area infiltrated in evaluated hpf) | No infiltrate | 0 | |
|  |  | Minimal acute (<10%) | 0.25 | |
|  |  | Mild chronic (10-25%, scattered neutrophils) | 0.5 | |
|  |  | Moderate chronic (26-50%) | 0.75 | |
|  |  | Marked (>51%, dense infiltrate) | 1 | |
|  | Extent (expansion of leukocyte infiltration) | Mucosal | 0.5 | |
|  |  | Mucosal and submucosal | 0.75 | |
| **Epithelial changes** | Hyperplasia (increase in epithelial cell numbers in longitudinal crypts, visible as crypt elongation) | No hyperplasia | 0 | |
|  |  | Minimal (<25%) | 0.25 | |
|  |  | Mild (26-35%) | 0.5 | |
|  |  | Moderate (36-50%, mitoses in the upper third of the crypt epithelium) | 0.75 | |
|  |  | Marked (>51%, mitoses in crypt epithelium distant from crypt base) | 1 | |
|  | Goblet cell loss (reduction of goblet cell numbers relative to baseline goblet cell numbers per crypt) | No loss | 0 | |
|  |  | Minimal (<25%) | 0.25 | |
|  |  | Mild (26-35%) | 0.5 | |
|  |  | Moderate (36-50%) | 0.75 | |
|  |  | Marked (>51%) | 1 | |
| **Mucosal architecture** | Ulceration (epithelial defect reaching beyond muscolaris mucosae) | No ulcers | 0 |  |
|  |  | Ulcers | 0.25 |  |
|  | Granulation tissue (connective tissue repair with new capillaries, surrounded by infiltrating cells, hypertrophied areas) | No granulation tissue | 0 |  |
|  |  | Granulation tissue | 0.25 |  |
|  | Mucosal thickness and crypt depth | No thickening | 0 |  |
|  |  | Thickening | 0.5 |  |
|  | Glandular rarefaction | No rarefaction | 0 |  |
|  |  | Rarefaction | 0.5 |  |
|  | Dysplasia | No dysplasia | 0 |  |
|  |  | Dysplasia | 0.5 |  |
|  |  | **MAX SCORE** | **6** |  |
